# A *PALB2 *mutation associated with high risk of breast cancer

**DOI:** 10.1186/bcr2796

**Published:** 2010-12-23

**Authors:** Melissa C Southey, Zhi L Teo, James G Dowty, Fabrice A Odefrey, Daniel J Park, Marc Tischkowitz, Nelly Sabbaghian, Carmel Apicella, Graham B Byrnes, Ingrid Winship, Laura Baglietto, Graham G Giles, David E Goldgar, William D Foulkes, John L Hopper, kConFab

**Affiliations:** 1 Department of Pathology, The University of Melbourne, Victoria 3010, Australia; 2 Centre for Molecular, Environmental, Genetic and Analytic Epidemiology, The University of Melbourne, Victoria 3010, Australia; 3 Program in Cancer Genetics, Departments of Oncology and Human Genetics, McGill University, Montréal, QC, H2W 1S6, Canada; 4 Segal Cancer Centre, Lady Davis Institute, Jewish General Hospital, Montréal, QC, H2W 1S6, Canada; 5 The International Agency for Research on Cancer, 150 Cours Albert Thomas, F-69372 Lyon CEDEX 08, France; 6 Department of Medicine, University of Melbourne, and Royal Melbourne Hospital, Parkville, Victoria 3050, Australia; 7 Cancer Epidemiology Centre, The Cancer Council Victoria, Rathdowne Street, Carlton 3052, Australia; 8 Department of Dermatology, University of Utah School of Medicine, Salt Lake City, UT 84112, USA; 9 The Peter MacCallum Cancer Centre, St Andrews Place, East Melbourne, Victoria 3002, Australia

## Abstract

**Introduction:**

As a group, women who carry germline mutations in partner and localizer of breast cancer 2 susceptibility protein (*PALB2*) are at increased risk of breast cancer. Little is known about by how much or whether risk differs by mutation or family history, owing to the paucity of studies of cases unselected for family history.

**Methods:**

We screened 1,403 case probands for *PALB2 *mutations in a population-based study of Australian women with invasive breast cancer stratified by age at onset. The age-specific risk of breast cancer was estimated from the cancer histories of first- and second-degree relatives of mutation-carrying probands using a modified segregation analysis that included a polygenic modifier and was conditioned on the carrier case proband. Further screening for *PALB2 *c.3113G > A (W1038X) was conducted for 779 families with multiple cases of breast cancer ascertained through family cancer clinics in Australia and New Zealand and 764 population-based controls.

**Results:**

We found five independent case probands in the population-based sample with the protein-truncating mutation *PALB2 *c.3113G > A (W1038X); 2 of 695 were diagnosed before age 40 years and 3 of 708 were diagnosed when between ages 40 and 59 years. Both of the two early-onset carrier case probands had very strong family histories of breast cancer. Further testing found that the mutation segregated with breast cancer in these families. No c.3113G > A (W1038X) carriers were found in 764 population-based unaffected controls. The hazard ratio was estimated to be 30.1 (95% confidence interval (CI), 7.5 to 120; *P *< 0.0001), and the corresponding cumulative risk estimates were 49% (95% CI, 15 to 93) to age 50 and 91% (95% CI, 44 to 100) to age 70. We found another eight families carrying this mutation in 779 families with multiple cases of breast cancer ascertained through family cancer clinics.

**Conclusions:**

The *PALB2 *c.3113G > A mutation appears to be associated with substantial risks of breast cancer that are of clinical relevance.

## Introduction

An increasing number of so-called moderate-risk [1] breast cancer susceptibility genes have been identified. Protein-truncating mutations in ataxia telangiectasia mutated (*ATM*), breast cancer type 1 susceptibility protein (BRCA1)-interacting protein 1 (*BRIP1*) and partner and localizer of the breast cancer 2 early onset protein (BRCA2) (*PALB2*), as well as CHK2 checkpoint homolog (*CHEK2*), have been observed to be nearly 10 times more likely in patients with strong breast cancer family histories than in unaffected controls [2-4]. The implications of these observations are not straightforward, so it is difficult to use the cancer histories of these families to make informative estimates of risk (penetrance) when the reason for studying them in the first place is their family cancer history [2-5]. Inference is more informative when based on testing families unselected for family history, but there is a paucity of such data for these genes.

Predicated on the assumption that the increased risk associated with a mutation multiplies a woman's underlying familial genetic risk, for these genes it has been estimated that rare protein-truncating mutations are associated with, on average, two- to threefold increases in risk [1-4]. Therefore, this increased risk must be interpreted by taking into account a carrier's family history. Consequently, for a given gene, the absolute risk is not the same for all carriers of such mutations [5].

It is also possible that the increased risk is not the same for all mutations. Some mutations in moderate-risk genes are high risk. For example, population-based studies have shown that the 7217T > G variant in *ATM *is associated with a substantially increased risk comparable to that for mutations in *BRCA1 *and *BRCA2 *[6], as has a subset of rare substitutions in *ATM *[7]. Another example is the Finnish founder *PALB2 *mutation; on the basis of studying cases unselected for family history, it has been estimated to be associated with a sixfold increased risk and a cumulative breast cancer risk of 40% by age 70 years [8].

We tested Australian women affected and unaffected by breast cancer and selected and unselected for family history for *PALB2 *mutations to estimate the prevalence and penetrance of these mutations in the Australian population.

## Materials and methods

### Participants

The Australian Breast Cancer Family Registry (ABCFR) is a population-based, case control family study of breast cancer with an emphasis on early-onset breast cancer that is being carried out in Melbourne and Sydney, Australia [9-11], and is part of the Breast Cancer Family Registry [12]. All adult women living in the metropolitan areas of Melbourne and Sydney who were diagnosed with a histologically confirmed first primary cancer of the breast were invited to participate in the ABCFR. From January 1, 1992, through September 30, 1999, in Melbourne and from January 1, 1993, through December 31, 1998, in Sydney, women younger than 40 years of age at diagnosis were selected; after January 1, 1996, random samples of women ages 40-49 years and 50-59 years at diagnosis were selected. All recruitment was done regardless of family history.

Cases were identified by use of the Victoria and New South Wales cancer registries, to which notification of cancer diagnoses is a legislative requirement. Overall, we approached 2,303 eligible individuals (ages at diagnosis: <40 years, 1,208; 40-49 years, 551; 50-59 years, 544), which resulted in 1,610 cases, and blood samples were collected from 94% of these cases. Cancer in relatives was verified by cancer registry reports, medical records or death certificates.

Previous mutation screening performed on the germline DNA of these women included screening for *BRCA1*, *BRCA2*, *TP53 *and *ATM *using a variety of mutation detection techniques [13-20]. These identified *BRCA1 *(*n *= 47), *BRCA2 *(*n *= 48), *ATM *(*n *= 1) and tumor protein 53 (*TP53*) (*n *= 5) mutation carriers who were not excluded from *PALB2 *mutation screening performed in this study. Six hundred ninety-five women under the age of 40 years diagnosed with breast cancer were available from the ABCFR for high-resolution melting (HRM) curve analysis of *PALB2*, and 708 women ages 40 years and over and diagnosed with breast cancer were screened for *PALB2 *c.3113G > A using the Custom TaqMan SNP Genotyping Assay (Applied Biosystems, Carlsbad, CA, USA). The study was approved by the ethics committees of The University of Melbourne and The Cancer Council Victoria, and all participants provided written informed consent for participation in the study.

The Kathleen Cuningham Foundation Consortium for Research in Familial Breast Cancer (kConFab) collected the same epidemiological, family history and lifestyle data, as well as biospecimens, as the ABCFR from more than 1,500 Australasian families with multiple cases of breast encompassing 10,000 individuals gathered from family cancer clinics in Australia and New Zealand [21]. For 778 families we obtained a DNA sample from the youngest affected member who had provided a blood sample and who had been tested and found not to carry a *BRCA1 *or *BRCA2 *mutation. All participants provided written informed consent for participation in the study.

### High-resolution melting curve analysis and sequencing analysis of *PALB2*

The *PALB2 *genomic sequence was obtained from the National Center for Biotechnology Information (reference sequence number NG_007406.1). Primers (Geneworks, Hindmarsh, South Australia, Australia) (Additional file 1) were designed by using Primer3 software (Whitehead Institute and Howard Hughes Medical Institute, Cambridge, MA, USA). For optimal performance of the HRM curve analysis, primers were designed to amplify DNA products between 100 and 310 bp. A total of 35 fragments were designed to cover the coding and flanking intronic regions of *PALB2*. The primer sequences and annealing temperatures are listed in Additional file 2. Initially, 96 DNA were Sanger-sequenced as described in Tischkowitz *et al*. [22]. These data and the corresponding DNA were then used to establish the optimal conditions for HRM curve analysis. DNA extracted from peripheral blood samples provided by 1,473 case probands (695 from women diagnosed with breast cancer under the age of 40 years participating in the ABCFR and 778 from kConFab) were screened for germline *PALB2 *mutations using HRM curve analysis [23,24]. DNA was then systematically screened using this established method. HRM reactions were carried out in 15-μL volumes and included 1.5 μL of 10 × polymerase chain reaction (PCR) buffer (Applied Biosystems, Victoria, Australia), a 3 mM final concentration of MgCl_2 _(Applied Biosystems), a 100 μM final concentration of deoxyribonucleotide triphosphate (dNTP) (Bioline, Alexandria, New South Wales, Australia), a 200 nM final concentration of each primer (Geneworks) (Additonal File 2), a 2.3 μM final concentration of Syto9 (Invitrogen, Victoria, Australia), 0.25 U of AmpliTaq Gold (Applied Biosystems) and 3 μL of Q solution (Qiagen, Victoria, Australia). Each reaction underwent a hold of 10 minutes at 95°C and 40 cycles of amplification of 30 s at 95°C and 1 minute at annealing temperature followed by melting to dissociate double-stranded DNA. The temperature range for melting was set at ± 10°C of the melting temperature of each amplicon with a rise in temperature of 0.05°C/s. HRM analysis was performed using Rotor-Gene 6000 Series Software 1.7 (Qiagen). Fragments displaying aberrant melt curves were sequenced to determine potential underlying genetic variations. For sequencing reactions, we utilized larger amplicons than those generated during HRM curve analysis (Additional file 1). Sequencing was carried out in 10-μL reactions, which included 1 μL of 10 × PCR buffer (Applied Biosystems), a 3 mM final concentration of MgCl_2 _(Applied Biosystems), a 100 μM final concentration of dNTP (Bioline), a 200 nM concentration of each primer (Geneworks), 0.25 U of AmpliTaq Gold (Applied Biosystems) and 3 μL of Q solution (Qiagen). PCR products were purified and analyzed on a 3130xl Genetic Analyser (Applied Biosystems) and the results were viewed using Chromas 1.45 (Technelusium, Tewantin, Queensland, Australia).

### TaqMan assays

Using the methods of Orlando *et al*. [25] and Ratnasinghe *et al*. [26], DNA extracted from the peripheral blood of 708 ABCFR probands diagnosed with breast cancer over the age of 40 years, 403 probands selected from the kConFab resource and 764 unaffected population controls from the ABCFR were analyzed for *PALB2*, c.3113G > A using the TaqMan assay. Each 10-μL reaction contained 5 μL of 2 × TaqMan Genotyping Master Mix (Applied Biosystems) and 0.125 μL of 40 × Custom Taqman SNP Genotyping Assay mix (Applied Biosystems), and the reactions were performed using a LightCycler 480 SW1.5 (Roche, Penzberg, Germany).

### Statistical methods for penetrance analyses

The age-specific hazard ratio (HR) for breast cancer (that is, the ratio of the age-specific breast cancer incidence rates for carriers of the mutation to that for noncarriers) was estimated using modified segregation analysis [27]. Models were fitted by the method of maximum likelihood using the statistical package MENDEL version 3.2 [28] (Department of Human Genetics, UCLA School of Medicine, Los Angeles, California, USA). To adjust for ascertainment, the likelihood for each pedigree was conditioned on the proband's phenotype (breast cancer status and age of onset) and genotype. Each pedigree involved data for the carrier proband and her first- and second-degree relatives.

A mixed model was employed which incorporates an unmeasured polygenic factor to model the effect on breast cancer risk of a large number of unmeasured genes in addition to the measured major gene [29]. The polygenic part of this model was implemented via a hypergeometric polygenic model with four loci [30] and postulates a normally distributed random variable *G *for each person so that these variables are correlated within families (see section 8.9 of Lange *et al*. [31]). A woman's age at breast cancer diagnosis was modeled as a random variable whose HR was, for noncarriers, exp(*G*) times the Australian breast cancer incidence rate for 1992-2002 [32] or, for carriers, the product of this HR multiplied by the age-specific HR. As in Antoniou *et al*. [33], the variance of *G *was chosen to be 1.67, and the mean was chosen so that the average HR for noncarriers equaled the population incidence. When testing for an age dependence of the HR, the model with a constant HR was compared to one where the HR was a continuous, piecewise linear function of age which was constant before age 40 years, linear between ages 40 and 60 years and constant after age 60 years.

All models assumed Hardy-Weinberg equilibrium at the *PALB2 *locus, a dominant action of *PALB2 *c.3113 G > A on breast cancer risk, conditional independence of all phenotypes given genotypes and an allele frequency of 0.001 for the variant in the Australian population. Two-sided *P *values for the modified segregation analyses were based on the likelihood ratio test.

Age-specific cumulative risk estimates were calculated from these HRs as one minus the exponential value of the cumulative HR, and the corresponding confidence intervals were calculated using a parametric bootstrap with 5,000 replications. More specifically, 5,000 draws were taken from the normal distribution that the parameter estimates would be expected to follow under asymptotic likelihood theory. For each age, corresponding values of the cumulative risk were calculated, and the 95% CI was taken to be the 2.5th and 97.5th percentiles of this sample. To test the accuracy of this estimate, we compared an appropriate weighted average of the corresponding survival functions (which are one minus the cumulative risk) with the Kaplan-Meier survival curve [33].

## Results

**Figure 1:**
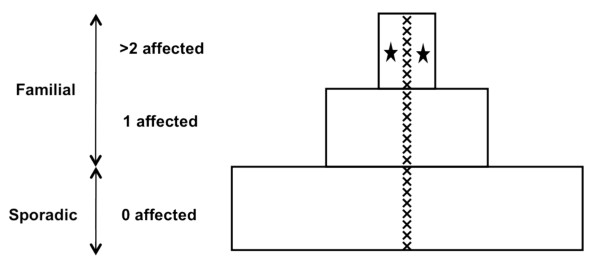
**Women diagnosed with breast cancer under age 40 years: The Australian Breast Cancer Family Registry**. The majority of breast cancer patients diagnosed before the age of 40 years have no affected family members (zero affected; the "sporadic" breast cancer group), some probands have one affected family member (one affected), and ~8% have a stronger family history (the "familial" breast cancer group). A strong family history is defined as the case proband having two or more first- or second-degree relatives affected by breast or ovarian cancer. x represents the 22 partner and localizer of the breast cancer 2 early onset protein (*BRCA2*) mutation carriers identified by previous testing in the ABCFR [13,19]. The filled black stars represent the two probands found to carry *PALB2 *c.3113G > A who both have a very strong family history of breast cancer. Adapted from Hopper [42].

We tested for *PALB2 *mutations using HRM curve analysis to scan DNA extracted from peripheral blood samples taken from a sample unselected for family history of women diagnosed with breast cancer before the age of 40 years (probands) from a population-based case control family study (ABCFR). Two of the 695 probands diagnosed before the age of 40 years were found to carry the *PALB2 *c.3113G > A (W1038X) mutation, and both had strong family histories of breast cancer. Figure 1 shows that only 8% of all tested probands had two or more first- or second-degree relatives with breast or ovarian cancer [13], yet both these carriers were in this extreme group.

**Figure 2:**
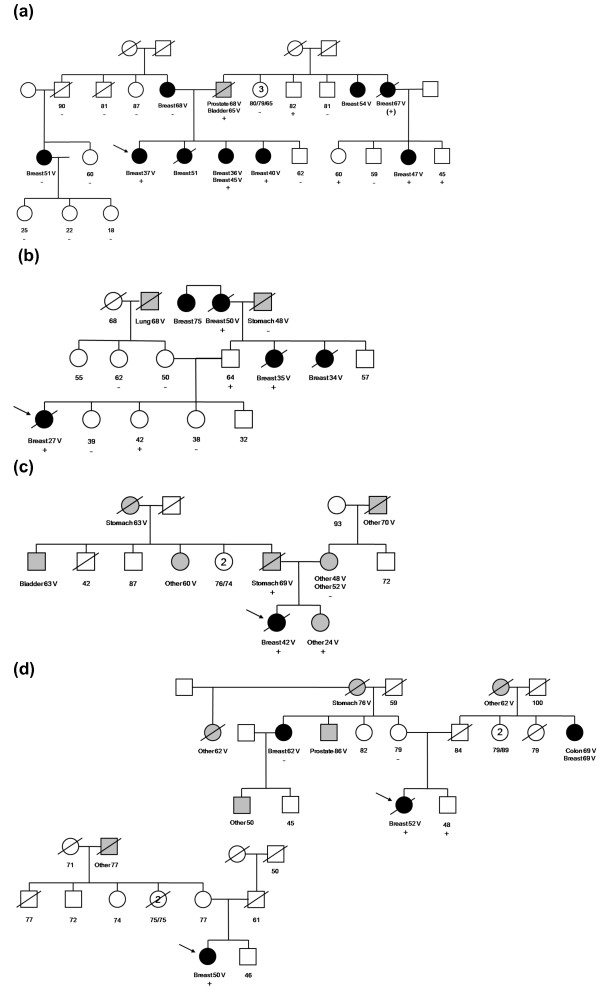
**Pedigrees of the *PALB2 *c.3113G > A mutation-carrying families**. Pedigrees of the *PALB2 *c.3113G > A mutation carrying families with **(a) **probands (indicated by arrows) and **(b) **families with probands diagnosed under the age of 40 years identified in the population-based study. **(c) **Family with probands diagnosed under the age of 50 years and **(d) **families diagnosed under the age of 60 years. Breast cancer is indicated by black filled symbols, and other cancers are indicated by gray filled symbols. All primary cancer diagnoses are indicated for each individual. Numbers within symbols represent multiple individuals. Breast, breast cancer; V, cancer verified; +, *PALB2 *c.3113G > A-positive; -, *PALB2 *c.3113G > A-negative; (+), obligate carrier;/, deceased.

Proband 1 was diagnosed at the age of 37 years with a grade 3 infiltrating ductal carcinoma. Figure 2a shows that proband 1 had three sisters who had all been diagnosed with breast cancer (one had two primary diagnoses) at the ages of 36, 40, 45 and 51 years. Her mother had been diagnosed with breast cancer at the age of 68 years, and a maternal cousin had a breast cancer diagnosis at the age of 51 years. The proband's father had been diagnosed with bladder cancer at the age of 65 years and prostate cancer at the age of 68 years. Two paternal aunts had breast cancer diagnoses at ages 67 and 54 years, and a paternal cousin (the daughter of an affected paternal aunt) had been diagnosed with breast cancer at the age of 47 years. Predictive testing was performed for this *PALB2 *mutation using blood samples collected from 22 family members. Four affected female relatives were found to carry this mutation (one was an obligate carrier) that involved the paternal lineage. The proband's mother, maternal uncle and a maternal cousin with breast cancer did not carry the mutation.

Proband 2 was diagnosed with a grade 3 infiltrating ductal carcinoma at the age of 27 years. Figure 2b shows that she had two paternal aunts diagnosed with breast cancer at ages 34 and 35 years and a paternal grandmother diagnosed at the age of 50 years (all deceased). Predictive testing was performed for this *PALB2 *mutation using blood samples collected from seven family members as well as paraffin-embedded tissue from three of the affected relatives (one paternal aunt and two paternal grandparents). The proband's father and two affected female relatives related to the father carried the mutation. One unaffected sister of the proband, age 42 years at last contact, carried *PALB2 *c.3113G > A. The proband's mother, a maternal aunt and two sisters, all unaffected, did not carry the mutation.

We then used a TaqMan assay to screen women diagnosed with breast cancer between the ages of 40 and 59 years and control probands participating in the ABCFR for the *PALB2 *c.3113G > A mutation. One of the 360 probands diagnosed at age 40-49 years and 2 of 348 diagnosed at ages 50-59 years were found to carry the mutation. Figures 2c-2d show that all of these carriers had a family cancer history, and one included verified reports of breast cancer in relatives. None of 764 tested population-based controls carried this mutation.

**Figure 3:**
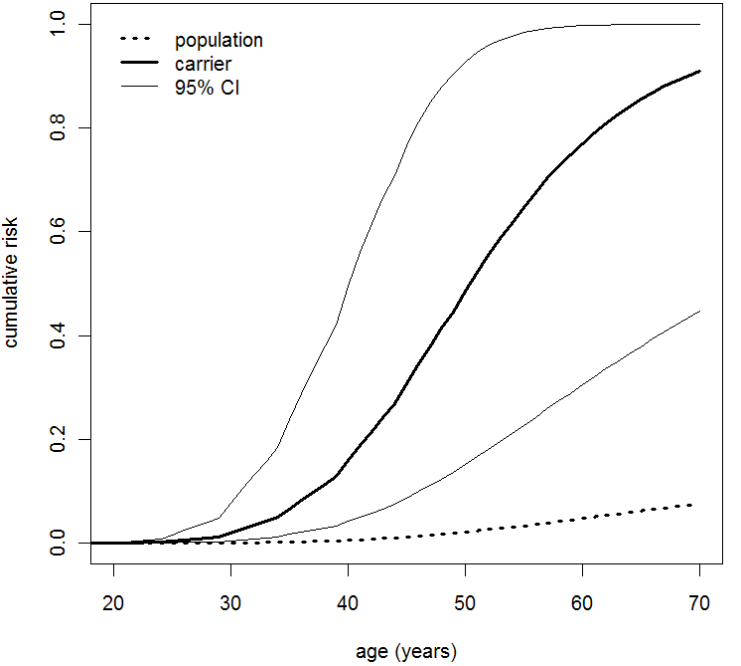
**Age-specific cumulative risks for *PALB2 *c.3113G > A mutation carriers**. Age-specific cumulative risks of breast cancer for women carrying the *PALB2* c.3113G > A mutation (unbroken lines) and for women in the general Australian population (dotted line).

**Figure 4:**
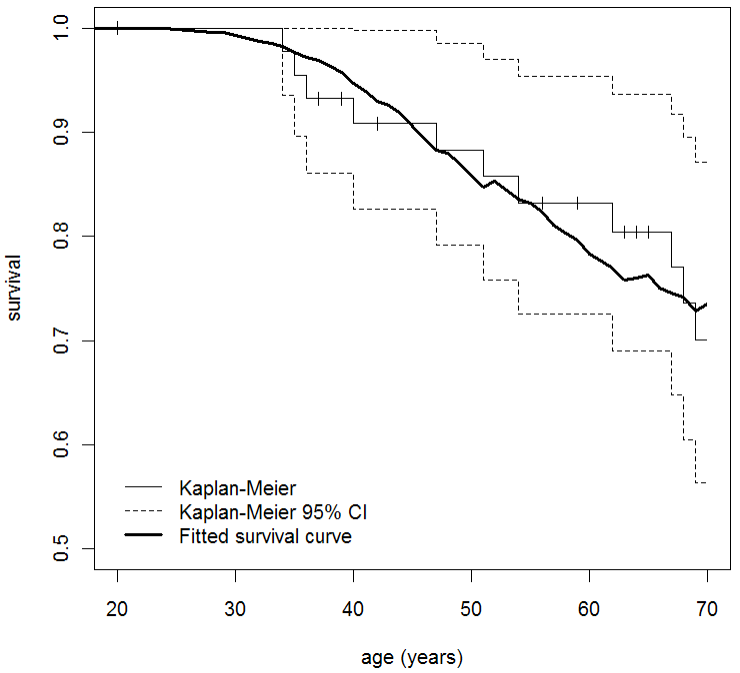
**The Kaplan-Meier survival curves for *PALB2 *c.3113G > A mutation carriers**. The Kaplan-Meier survival curve with breast cancer as the outcome for female first- and second-degree relatives of the probands with the fitted survival curve overlaid. The fitted survival curve was calculated as the average of the survival curves for carriers and noncarriers, each of these being equal to the exponential minus the appropriate cumulative incidences. For the averaging of survival curves, we used age-specific weights equal to the expected proportion of relatives at risk of breast cancer at a given age who carry (for the carrier survival curve) or do not carry (for the noncarrier survival curve) the partner and localizer of breast cancer 2 susceptibility protein (*PALB2*) mutation. Carrier probabilities for the relatives were calculated from known genotypes and family relationships, but not from affected status or other phenotypes, using a modified version of Mendel 3.2 software [28].

Using the five population-based mutation carrier families, we estimated the breast cancer HR using the cancer histories of first- and second-degree relatives and found it to be 30.1 (95% CI, 7.5-120; *P *< 0.0001), independent of age (*P *= 0.8; though because of low power, a modest age dependence cannot be ruled out). These data corresponded to estimated age-specific cumulative risk ratios (penetrance) of 49% (95% CI, 15-93) to age 50 years and 91% (95% CI, 44-100) to age 70 years (see Figure 3). Our confidence in these estimates was enhanced by their high degree of agreement with the Kaplan-Meier survival curves for female first- and second-degree relatives of the probands, which accounted for the fact that these relatives are a mix of carriers and noncarriers (see Figure 4). The HR corresponding to the *PALB2 *W1038X variant could not be distinguished from the average of the age-specific HRs for *BRCA2 *mutations reported by Antoniou *et al*. [34] of 13.6 (*P *= 0.3). It was higher than a HR of 2.3, which is roughly equivalent to the odds ratio of 2.3 for *PALB2 *variants reported by Rahman *et al*. [4] (*P *= 0.0003).

HRM screening for *PALB2 *mutations for 779 families with multiple cases of breast cancer recruited through Australian and New Zealand clinics by the kConFab consortium identified a further eight (1.0%) families that carried the *PALB2 c*.3113G > A mutation. We did not attempt to estimate penetrance using these family histories, since they were nonsystematically selected by their family histories of cancer.

## Discussion

Studies conducted in the United Kingdom, Finland, Italy, Spain and Canada have shown that, while rare, mutations in *PALB2 *are far more common in breast cancer patients with a strong family history than in unaffected population-based controls. In the UK study, 10 (1.1%) of 923 strongly familial cases, compared with 0 of 1,084 unaffected controls, were mutation carriers (*P *= 0.004) [4]. The Finnish recurrent mutation *PALB2 *(c.1592delT) was carried by 3 (2.7%) of 113 familial breast cancer cases compared with 6 (0.2%) of 2,501 controls, an 11-fold difference (95% CI, 3-44) [35]. A French-Canadian founder mutation, c.2323C > T, resulting in Q775X, was found in 2 (0.6%) of 356 unselected early-onset cases compared with 1 (2%) of 50 familial cases and none (0%) of 6442 newborns [36]. Papi *et al*. [37] screened 132 Italian families with breast cancer without *BRCA1 *or *BRCA2 *mutations and identified one protein-truncating mutation (c.2257C > T, R753X) that was not observed in 300 control DNA. Garcia *et al*. [38] reported one protein-truncating mutation (c.1056-1057delGA, K3531X) in a screen of 95 families with multiple cases of breast cancer without *BRCA1 *or *BRCA2 *mutations. These observations, while showing that *PALB2 *mutations are associated with an increased risk of breast cancer, cannot be used to obtain direct or precise estimates of the magnitude of increased risk [4,5].

Estimation of the average increased risk associated with *PALB2 *mutations has been performed previously using an indirect method by presuming a polygenic modifier model. This method estimated that, on average, *PALB2 *protein-truncating mutations conferred a 2.3-fold increased risk of breast cancer (95% CI, 1.4-3.9; *P *= 0.0025) [4]. For one specific Finnish founder mutation, a direct estimate was made from a sample of population-based cases unselected for family history and screened for the *PALB2 *1592delT mutation [8]. Eighteen (0.9%) of the 1,918 cases carried the mutation. It was estimated from their family histories that carriers in this setting are at about six times the general population risk (95% CI, 2-17; *P *= 0.01), equivalent to a breast cancer risk of 40% by age 70 years (95% CI, 17-77), which is comparable to the 45% estimate found for *BRCA2 *mutation carriers in a large study of unselected carrier families [34].

In Australia, a *PALB2 *mutation has been found, c.3113G > A, carried by 5 (0.4%) of 1,403 unselected population-based patients diagnosed before age 60 years, 8 (1%) of 779 cases from families with multiple cases of breast cancer, and none of 764 unaffected population-based controls. This mutation was found twice in a UK study [4] that screened 923 cases from families with multiple cases of breast cancer. The Australian carrier families reported here are predominantly of Australian, English and Scottish heritage. These are the only reports of this mutation we know of, so it is possible that this mutation originated in the United Kingdom.

We estimated from analyses of case carrier families that carriers of the *PALB2 c*.3113G > A mutation who are relatives of unselected case carriers have a high risk of developing breast cancer: about 50% to age 50 years and 90% to age 70 years. The lower bound of the 95% CI of the latter estimate is about the same as the average risk for *BRCA2 *mutation carriers estimated using the same design and statistical methodology [39]. Therefore, this mutation is associated with as high a risk as other mutations being tested for by cancer family genetics services across the world.

For families with multiple cases of breast cancer currently attending cancer genetics services in Australia, at most 20% of those screened for *BRCA1 *and *BRCA2 *are found to carry mutations [21], which is similar to the rates in the United Kingdom [40] and the United States [41]. Although *PALB2 *c.3113G > A is rare, testing for it is inexpensive and has clinical utility. By virtue of being based on case families, our risk estimate applies to women with at least some family history, so it is appropriate for counseling carriers identified in families with multiple cases of breast cancer who use cancer family genetics services.

## Conclusions

Given that carriers of this *PALB2 *mutation appear to be at least at the conventional level of high risk, thought to apply to *BRCA1 *and *BRCA2 *mutation carriers, testing would seem justified in a clinical genetics setting. For the women and their family members who carry these mutations, it is potentially important that they be identified so that they can be offered appropriate prevention, screening and clinical management. There may be other *PALB2 *mutations that are also as highly penetrant, and this paradigm might apply to other genes, such at *ATM*, *BRIP1 *and *CHEK2*, for which mutations appear to be associated, on average, with moderately increased risks, but for which some mutations might be associated with high risk. Targeted clinical testing of such high-risk mutations in these genes could be justified.

## Abbreviations

ABCFS: Australian Breast Cancer Family Study; HR: hazard ratio; HRM: high-resolution melting; kConFab: The Kathleen Cuningham Foundation Consortium for Research in Familial Breast Cancer.

## Competing interests

The authors declare that they have no competing interests.

## Authors' contributions

MCS led this study, including the conception, design, acquisition of data, analysis, interpretation of data and preparation of the manuscript. ZLT conducted molecular analyses and contributed to the preparation of the manuscript. JGD led aspects of the analyses and contributed to the preparation of the manuscript. FAO conducted molecular analyses and contributed to the preparation of the manuscript. DJP provided critical input to the molecular analysis and manuscript preparation. MT conducted molecular analyses and contributed to the preparation of the manuscript. NS conducted molecular analyses and contributed to the preparation of the manuscript. GBB contributed to aspects of the analyses and contributed to the preparation of the manuscript. CA contributed data from the Australian Breast Cancer Family Study (ABCFS) resource and contributed to the preparation of the manuscript. IW provided critical input to the molecular analysis and manuscript preparation. LB led aspects of the analyses and contributed to the preparation of the manuscript. GGG, cofounder of the ABCFS resource, contributed data from the ABCFS and contributed to the preparation of the manuscript. DEG provided critical input to the molecular analysis, statistical analyses and manuscript preparation. WDF conducted molecular analyses and contributed to the preparation of the manuscript. JLH, cofounder of the ABCFS resource, contributed data from the ABCFS, contributed to analyses and the preparation of the manuscript. All authors read and approved the final manuscript.

## Supplementary Material

Additional file 1 **Sequencing primer sequences**. Primers designed for DNA sequencing, including DNA fragment size (bp) and annealing temperatures. Click here for file

Additional file 2 **Polymerase chain reaction primer sequences and annealing temperatures**. The primer sequences and annealing temperatures designed to amplify 35 fragments (including the coding and flanking intronic regions) of partner and localizer of breast cancer 2 susceptibility protein (*PALB2*). Click here for file
