# Supplementary material for: A PALB2 mutation associated with high risk of breast cancer
Source: Breast Cancer Res. 2010 Dec 23;12(6):R109. doi: 10.1186/bcr2796 (PMC3046454; doi:10.1186/bcr2796)
Supplement: Additional file 1 — Sequencing primer sequences. Primers designed for DNA sequencing, including DNA fragment size (bp) and annealing temperatures. [file bcr2796-S1.PDF]

Additional file 1 : Sequencing primer sequences

Exons	Name of primers	Sequence of the primers for HRM	Fragment size	Annealing Tm
**1**	ex1-F	GGATTTAATTGGCCGGAGTT	309	61˚C
	ex1-R	GACACAAAGCCAGGCCTAAA		
**2**	ex2A-F	GACTCCACCTTTCCACTTGC	104	60˚C
	ex2A-R	CGGGCTAGTGTCTTGCTGTA		
	ex2B-F	AAAGGAGAAATTAGCATTCTTGAAAA	161	60˚C
	ex2-R	AACAGCCCCAGAAATACGTT		
**3**	ex3-F	AAACGTATTTCTGGGGCTGTT	222	60˚C
	ex3-R	CACACTGTGGGAAAAAGAACAA		
**4**	ex4A1-F	TTCATCTGCCTGAATGAAATG	198	60˚C
	ex4A1-R	CATCAAGATGGGTTTTGATGTG		
	ex4A2-F	TTACACATCAAAACCCATCTTG	106	60˚C
	ex4A2-R	GTAATCCTCCTGGGCCATCT		
	ex4B-F	TTGGGCCTGAGTCCTTTAAC	204	60˚C
	ex4B-R	TGAGTGAATCAGTGCCAAAGA		
	ex4C-F	TTGGCACTGATTCACTCAGATT	251	57.6˚C
	ex4C-R	TGTAGTCGCCCTGGTGAAAT		
	ex4D-F	GGTGTTGATACATTCCTAAGAAGACC	266	60˚C
	ex4D-R	TTAGAACTTGTGGGCAGTTGG		
	ex4E-F	CAGATAACCTCCTTGTAAATAAAGCTA	300	60˚C
	ex4E-R	CAGGCACTGTGCAAGAATGT		
	ex4F-F	GCAACCTCTCCTCTTTCTGC	320	60˚C
	ex4F-R	GCGGGAGAGCTGACTTTAGTT		
	ex4G-F	ACATGCACAGGACAACCAAG	200	61.3˚C
	ex4G-R	GAAGTTGGCAAAAGTGGTTCA		
	ex4H-F	CACCCCAGCATCAGATCATT	200	61.3˚C
	ex4H-R	AAGGAAGTGCCAGGCAAATA		
	ex5A-F	TTGTCTGTTTTGTTGGGTTTTG	234	61.3˚C
	ex5A-R	GAGGTCCAAAGTCTTCATCAGG		

5	ex5A-F	TTGTCTGTTTTGTTGGGTTTTG	234	61.3˚C
	ex5A-R	GAGGTCCAAAGTCTTCATCAGG		
	ex5B-F	AGGGATGGAATGCTGAGTTT	229	61.3˚C
	ex5B-R	CCTCCATTTCTGTATCCATGC		
	ex5C-F	GGGAAGCTGTATTTTTCCAGA	244	60˚C
	ex5C-R	TAAGATGGGGAAAGCAGGTG		
	ex5D-F	TTGCGCCTGATGATAATGAC	242	61.3˚C
	ex5D-R	CACTTGCAGGGTGGTATGTG		
	ex5E1-F	CACCCCAACTTGCTCATT	105	58.8˚C
	ex5E1-R	ACACTTGCAGGGTGGTATGTG		
	ex5E2-F	AGCCCAGCAAAACCACATAC	153	58.8˚C
	ex5E2-R	AGCTCCTGGCATGTGTTTCT		
	ex5E3-F	ACACCTCCACCCATTGAGTC	169	58.8˚C
	ex5E3-R	GCAAGCAAGTCATGCTGTTTA		
6	ex6-F	AGTGGGTAATGCAGGCAGAC	213	60˚C
	ex6-R	TGACTGAATTCTTTTCAGTTCATT		
7	ex7-F	TGCTTTGCATAAAACAGCACT	293	60˚C
	ex7-R	TGGTAAGCTGCCCATCTACA		
8	ex8-F	TGGAAAATCTGGATTAAACAAAAA	221	58.8˚C
	ex8-R	TGCACTTAAAACCAGCTGACA		
9	ex9A-F	ATTAAAAGGTTACTCCTCACATCAC	141	60˚C
	ex9A-R	CTCTTTGTCAGGCCAAGCAC		
	ex9B-F	GTGCTTGGCCTGACAAAGAG	225	60˚C
	ex9Seq-R	TGTTGATGCGGTACATGCTT		
10	ex10-F	CCTAGAGACTGCTTTAGTGCAAA	250	60˚C
	ex10-R	TTCACAACAACCCTGTAAAATTAG		
11	ex11A-F	TTTTCTGAATACTGGTTTGTTGGA	136	60˚C
	ex11A-R	GGCTTTGTGACAGACTGAAGC		
	ex11B-F	GGTCAACTCCTGAAAAAGATGC	130	60˚C
	ex11B-R	CACTTAATGAGACCAACAGTAACACA		
	ex12A1-F	GAGCCTATCGGTCATTGCTT	153	60˚C

12	ex12A1-F	GAGCCTATCGGTCATTGCTT	153	60˚C
	ex12A1-R	TCTTTGGCACAGGGATGACT		
	ex12A2-F	CCGAAATTAGGGGCTTCTCTT	118	60˚C
	ex12A2-R	CACGCTGAGAGTCGTCTTAGG		
	ex12B-F	GAAGCCCTGTGTTTCAGCTC	138	60˚C
	ex12B-R	TTTCAGAATGTCCCACCCATAGA		
13	ex13A0-F	TGGGAACATGGTTTTGACCT	158	56.4˚C
	ex13A0-R	GTCCAAATGGCAATTGTTC		
	ex13A1-F	TCTTCTTTGTATGCTATCAGGTTCCT	198	60˚C
	ex13A2-R	TTTTTGTCCAGCCAGCAAAT		
	ex13B-F	TTGTGAAATGGTCGGGTACA	250	60˚C
	ex13B-R	CATCCAAGATCAGTGGTGCT		
	ex13C-F	TGATTGCTTGTTTATGTCCAGA	220	60˚C
	ex13C-R	GGGAAACAATAACATGCCAAG		

Annealing Tm: Annealing Temperature, F: Forward primer, R: Reverse primer.
