# Supplementary material for: A PALB2 mutation associated with high risk of breast cancer
Source: Breast Cancer Res. 2010 Dec 23;12(6):R109. doi: 10.1186/bcr2796 (PMC3046454; doi:10.1186/bcr2796)
Supplement: Additional file 2 — Polymerase chain reaction primer sequences and annealing temperatures. The primer sequences and annealing temperatures designed to amplify 35 fragments (including the coding and flanking intronic regions) of partner and localizer of breast cancer 2 susceptibility protein (PALB2). [file bcr2796-S2.PDF]

Additional file 2: PCR primer sequences and annealing temperatures.

Exons	Name of primers	Sequence of the primers for Sequencing	Fragment size	Annealing Tm
**1**	ex1-F	CACTGCTCGGCCGTCTA	423	60˚C
	ex1Seq-R	ATTTTCTGTGCCCCCTCAG		
**2,3**	ex2A-F	GACTCCACCTTTCCACTTGC	453	60˚C
	ex3-R	CACACTGTGGGAAAAAGAACAA		
**4**	ex4A1-F	TTCATCTGCCTGAATGAAATG	280	60˚C
	ex4A2-R	GTAATCCTCCTGGGCCATCT		
	ex4A2-F	TTACACATCAAAACCCATCTTG	265	58.8˚C
	ex4B-R	TGAGTGAATCAGTGCCAAAGA		
	ex4B-F	TTGGGCCTGAGTCCTTTAAC	437	60˚C
	ex4C-R	TGTAGTCGCCCTGGTGAAAT		
	ex4C-F	TTGGCACTGATTCACTCAGATT	469	60˚C
	ex4D-R	TTAGAACTTGTGGGCAGTTGG		
	ex4D-F	GGTGTTGATACATTCCTAAGAAGACC	499	62.7˚C
	ex4E-R	CAGGCACTGTGCAAGAATGT		
	ex4F-F	GCAACCTCTCCTCTTTCTGC	449	60˚C
	ex4G-R	GAAGTTGGCAAAAGTGGTTCA		
	ex4G-F	ACATGCACAGGACAACCAAG	360	60˚C
	ex4H-R	AAGGAAGTGCCAGGCAAATA		
**5**	ex5A-F	TTGTCTGTTTTGTTGGGTTTTG	378	61.3˚C
	ex5B-R	CCTCCATTTCTGTATCCATGC		
	ex5C-F	AGGGATGGAATGCTGAGTTT	202	60˚C
	ex5DF-R	GTCATTATCATCAGGCGCAA		
	ex5BR-F	GCATGGATACAGAAATGGAGG	206	60˚C
	ex5C-R	TAAGATGGGGAAAGCAGGTG		
	ex5D-F	TTGCGCCTGATGATAATGAC	361	60˚C
	ex5E2-R	AGCTCCTGGCATGTGTTTCT		
	ex5E1-F	CACCCCAACTTGCTCATT	320	60˚C
	ex5E3-R	GCAAGCAAGTCATGCTGTTTA		

**6**	ex6-F	AGTGGGTAATGCAGGCAGAC	283	62.7˚C
	ex6Seq-R	CCAATCCAAATCTGTTTTTCTGA		
**7**	ex7Seq-F	GCTCTTTCTTTTCACCTGCAT	376	60˚C
	ex7-R	TGGTAAGCTGCCCATCTACA		
**8**	ex8Seq-F	AATAAAAAGAGTTTTCTGAGCCTTC	300	60˚C
	ex8-R	TGCACTTAAAACCAGCTGACA		
**9**	ex 9A-F	ATTAAAAGGTTACTCCTCACATCAC	346	60˚C
	ex9Seq-R	TGTTGATGCGGTACATGCTT		
**10**	ex10Seq-F	CACGTTTTCTGGGTTAGATTTTT	330	60˚C
	ex10-R	TTCACAACAACCCTGTAAAATTAG		
**11**	ex11A-F	TTTTCTGAATACTGGTTTGTTGGA	136	60˚C
	ex11A-R	GGCTTTGTGACAGACTGAAGC		
	ex11B-F	GGTCAACTCCTGAAAAAGATGC	130	60˚C
	ex11B-R	CACTTAATGAGACCAACAGTAACACA		
**12**	ex12Seq-F	GAAGCCAGAAGTCCAGTCCA	395	60˚C
	ex12B-R	TTTCAGAATGTCCCACCCATAGA		
**13**	ex13Seq-F	TCTGTCCAAAACTGCAACACAA	357	60˚C
	ex13A2-R	TTTTTGTCCAGCCAGCAAAT		
	ex13A1-F	TCTTCTTTGTATGCTATCAGGTTCCT	198	60˚C
	ex13A2-R	TTTTTGTCCAGCCAGCAAAT		
	ex131Seq-F	TCCTCCCACCTGTCTCTGAC	432	62.7˚C
	ex13C-R	GGGAAACAATAACATGCCAAG		

Annealing Tm: Annealing Temperature, F: Forward primer,

R: Reverse primer.
